# Chiral Transformation of a Nanostructured Silver Film by Illumination with Circularly Polarized Light

**DOI:** 10.1021/acsnano.6c00256

**Published:** 2026-03-04

**Authors:** Daler R. Dadadzhanov, Nikita S. Petrov, Igor A. Gladskikh, Daniel Feferman, Nikita A. Toropov, Leilei Gu, Peng Yu, Zhiming Wang, Tigran A. Vartanyan, Alexander O. Govorov, Gil Markovich

**Affiliations:** † Raymond and Beverly Sackler Faculty of Exact Sciences, School of Chemistry, 26745 Tel Aviv University, Tel Aviv 6997801, Israel; ‡ International Research and Education Centre for Physics of Nanostructures, 65071 ITMO University, St. Petersburg 197101, Russia; § 7423 University of Southampton, Southampton SO17 1BJ, U.K.; ∥ Department of Physics and Astronomy, Nanoscale and Quantum Phenomena Institute, 1354 Ohio University, Athens, Ohio 45701, United States; ⊥ Institute of Fundamental and Frontier Sciences, 12599 University of Electronic Science and Technology of China, Chengdu 610054, China

**Keywords:** localized plasmon resonance, circular dichroism, chirality, laser–matter interaction, metal nanoparticles, reshaping

## Abstract

Chiral plasmonic nanoparticles combine interesting geometrical and optical properties and hold great promise for applications in polarization optics, light sources, and enantioselective sensing. However, simple, low-cost, large-area, and reproducible routes to fabricate such chiral plasmonic nanostructures are still limited. Here, we demonstrate a scalable chiral optical imprinting of nanostructured Ag films in which an initially racemic ensemble of nanoparticle enantiomers is transformed into a chiral state under continuous-wave circularly polarized illumination. The imprinting is driven by plasmon-induced redox processes mediated by hot carriers and by surface diffusion of Ag^+^ ions and leads to a robust, unchanged circular dichroism upon flipping the sample, evidencing the formation of 3D chiral nanostructures. We identified two different irradiation regimes depending on the power density. At low-power densities, circularly polarized light creates asymmetrical distribution of electric field around nanoparticles that promotes the growth and stabilization of Ag nanoparticles with handedness matching the incident light polarization. The microscopic origin of the observed chiral growth was elucidated by numerical simulations of the near-field response and hot-carrier generation, which show that under circularly polarized illumination, size-asymmetric (achiral) Ag dimers act as plasmonic nanoantennas, giving rise to a strongly handed local hot-carrier generation profile. At high-power densities (>5 W·cm^–2^), selective hot-carrier-driven photo-oxidation and thermally assisted coarsening of same-handed particles dominate, giving rise to inversion of the enantiomeric excess. The described transformations are observed only in the aged granular films, where a thin Ag_2_O layer accelerates Ag atom/ion migration and facilitates nanoparticle reshaping. The resulting films exhibit a pronounced chiroptical response with a maximum *g*-factor of 1.2 × 10^–2^ at 532 nm.

## Graphical abstract

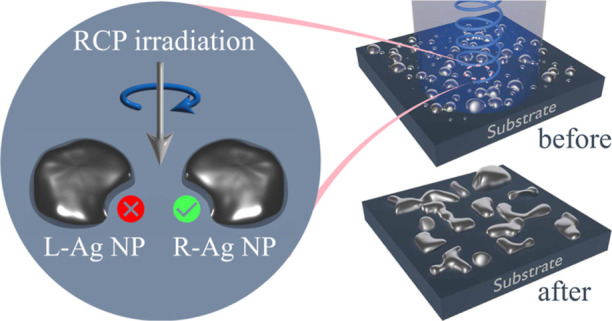

## Introduction

Chiral nanoparticles (NPs), as in the case of chiral molecular structures, cannot be superimposed with their mirror image. At the molecular level, the two mirror-image versions of a chiral structure are called enantiomers. Each enantiomer specifically interacts with circularly polarized light, meaning that the enantiomers can absorb differently the two circularly polarized states of light. This property, named circular dichroism (CD), has been used to study biomolecular conformations and conformation changes and helps determining enantiomeric excess in the mixtures of the two enantiomers.^1^ Typically, chiral plasmonic NPs are formed either by colloidal chemistry^2–4^ or physical methods such as focused ion beam milling (FIB),^5,6^ electron beam lithography (e-beam),^7–9^ and colloidal nanosphere^10,11^ lithography or glancing angle deposition of metal on seed particles while rotating the substrate.^12,13^ The formation of 2D chiral nanostructures with strong CD via FIB or e-beam lithographies is spatially limited by technical characteristics or energy consumption and the high cost of manufacturing.

Localized surface plasmon resonances in metal NPs can promote chirality via optical near-field enhancement, heat generation, and hot-charge carrier injection.^3,4,14–17^ Active debates within the scientific community revolve around the mechanism of plasmon induced chemical processes, whether they are caused by photothermal heating, the generation of hot charge carriers, or local field effects. Studies by Ding et al. have demonstrated light-induced reshaping of individual gold NPs from a spherical to a disk-like shape on a silicon surface with a thin TiO_2_ layer by using continuous wave (CW) laser illumination.^18^ Recently, Saito and Tatsuma proposed a light-induced method for creation of chiral nanostructures on a substrate^19^ based on plasmon-induced charge separation. Especially, chiral NPs are formed due to a redox process at the sites where electron–hole pairs are generated. The hot electrons from Au nanostructures were injected into the TiO_2_ substrate and hot holes caused an oxidation reaction of adsorbed Pb^2+^ ions and consequent deposition of PbO_2_. Demonstration of plasmon-induced charge separation was also reported for fabrication of chiral nanostructures via photoelectrochemical dealloying of Au–Ag alloy under circularly polarized light.^20^ Nonthermal reshaping of an ensemble of sodium NPs on a sapphire substrate by CW laser illumination at 875 nm was reported by Vartanyan et al.^21^ It was claimed that low-intensity illumination can induce the mass transfer of sodium atoms in only those NPs within the inhomogeneous ensemble that are resonant with the incident light. This phenomenon is evidenced by the appearance of a spectral hole at the excitation wavelength in the differential extinction spectra. More interestingly, UV-light illumination at an intensity of 20 mW/cm^2^ for prolonged time of a silver film reduces the dewetting rate and enhances the optical and morphological stability during thermal annealing, compared to untreated substrates.^21^ Similarly, the anisotropic optical properties of Ag and Au nanostructures can be controlled through thermal and/or laser dewetting.^22–24^ In this regard, we recently developed a method to create highly anisotropic plasmonic metasurfaces by leveraging the polarization and wavelength of linearly polarized laser light to selectively reshape isotropic granular metal films, resulting in linearly dichroic metasurfaces with permanent spectral holes. Motivated by the persistent spectral hole-burning technique, we demonstrated induced CD in Au nanostructures, achieved through pulsed laser illumination of achiral NPs embedded in porous matrices.^23^ However, to date, comprehensive studies on utilizing circularly polarized light (CPL) for inducing chirality in self-organized NPs remain limited and have not been widely reported.

Here, we demonstrate the transformation of a nanostructured Ag film (Ag NF) from achiral to chiral under CPL irradiation with relatively low-power densities. It was found that the observed CD band for the inhomogeneous Ag NF upon CPL illumination arises from plasmon-induced localized photo-oxidation of Ag^+^, leading to the reshaping of the Ag NF. The wavelength- and CPL handedness-dependent CD was studied by irradiation of achiral Ag NFs using CW lasers at 405 and 532 nm. Notably, aged Ag NFs, which are composed of polycrystalline grains and underwent restructuring under ambient conditions in humid air at room temperature, exhibited stronger CD than the fresh ones due to the presence of a Ag_2_O layer as a source for mobile Ag^+^ ions. Hence, the development of a facile, cost-effective, and large-scale method for production of chiral NFs via plasmonic photochemical effects opens up opportunities for advanced sensing, optically encrypted electronic applications, and high-performance CPL detectors.

## Results and Discussion

### Light-Induced Chirality in a Ag Nanostructured Film

The general idea of the reported chirality induction is that irradiation with CPL leads to the excitation of localized plasmon resonance through photon absorption only by resonant NPs (Figure 1a). We hypothesize that before irradiation, the NF consists of a racemic, irregular array of chiral Ag NPs whose chirality arises from random shape imperfections in both the lateral and vertical (height) directions. First, this intrinsic chirality is governed by the local shape asymmetries of individual NPs. On the other hand, illumination of closely spaced NPs with CPL can induce more complex, collective interactions between them so that an ensemble can be effectively regarded as a chiral NP cluster.^25^ In this case, induction of CD in the films can be explained both ways, either as breaking of the racemic balance of isolated NPs or of coupled NP clusters. Chiral assemblies have been extensively studied using DNA-assembled chiral plasmonic nanostructures consisting of coupled spherical NPs, and their behavior can be explained by the mechanical model of Born and Kuhn,^26,27^ which describes a coupled electron oscillator system. To gain insight into the conversion of racemic NF into imbalanced chiral ones, we introduce the labels **L-Ag NPs** and **D-Ag NPs** for the randomly chiral NPs/clusters. These labels correspond to structures that preferentially absorb left-handed circularly polarized (LCPL) or right-handed circularly polarized (RCPL) light, respectively.

**Figure 1:**
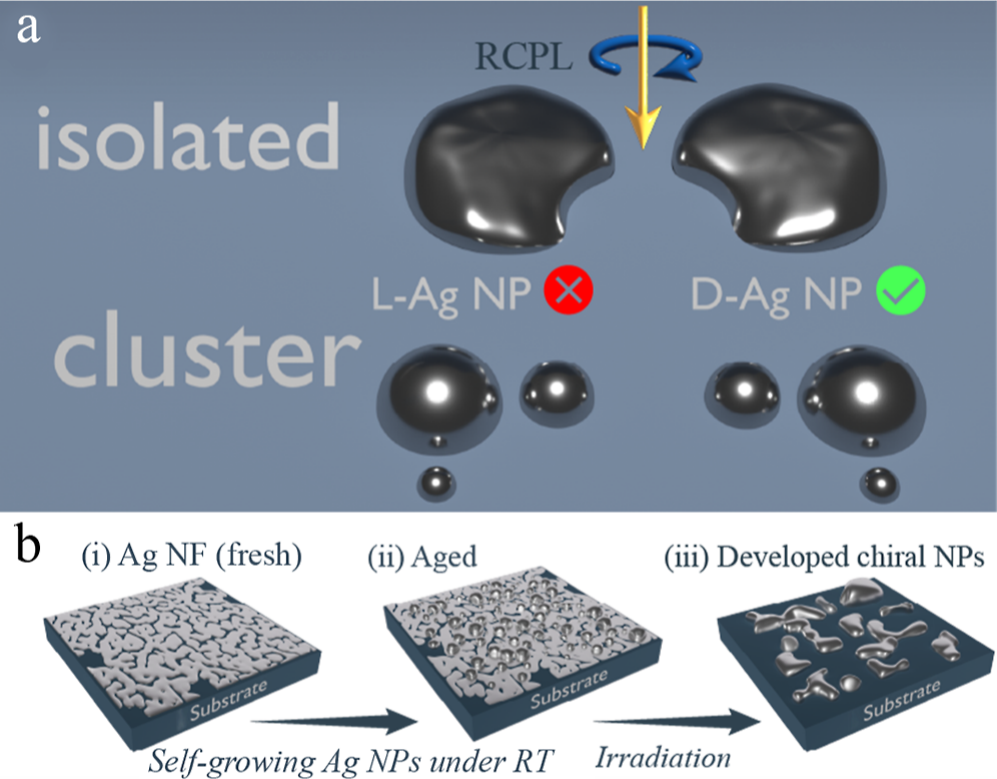
Schemes of (a) light-induced chirality mechanism in self-organized Ag NPs under excitation of isolated and cluster NPs with RCPL. The green check mark indicates that the D-Ag NP more effectively absorbs RCPL than the L-Ag NP. This preferential absorption may lead through various processes to an imbalance between right- and left-handed NPs, or NP clusters. (b) Scheme of Ag NF preparation.

Figure 1b presents the scheme of chiral Ag NP fabrication. First, the as-deposited (hereinafter referred to as “fresh”) Ag NFs are deliberately subjected to self-aging by storing in ambient air conditions for two months (named as “aged”). The aging effect led to the self-growth of partially oxidized Ag NPs on top of the Ag film (Figure 1b). The comparison of HR-SEM images of “fresh” and “aged” Ag NF is shown in Figure S1. This effect can be attributed to the surface diffusion of Ag ions originating in the Ag oxide coating formed on exposure to humid air, which triggers nucleation, followed by the formation of islands and the growth of individual NPs. The aged Ag NF consists of oblate NPs with an average diameter of 38 nm (Figure S2). Additionally, the HR-SEM image (Figures 2d and S1) reveals a background labyrinth-like structure, characteristic of an unannealed thin Ag NF. These morphological features are associated with the accelerated nucleation of Ag NPs, driven by the chemisorption of O_2_ from humid air.^28–30^

**Figure 2:**
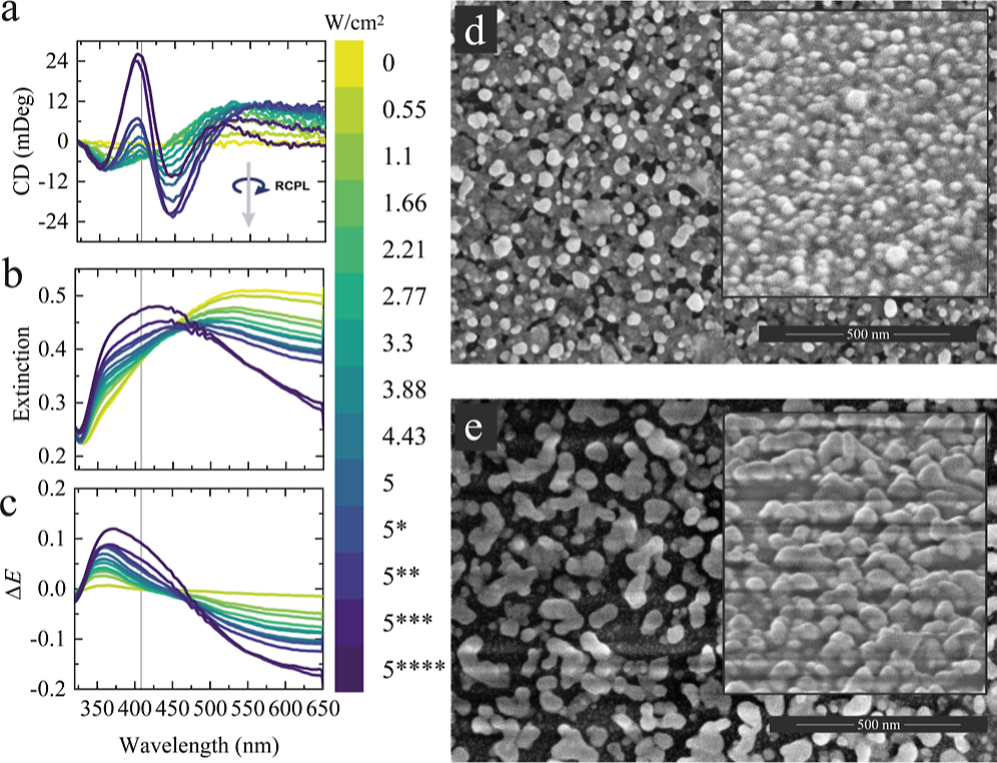
(a) CD, (b) extinction, and (c) differential extinction spectra (Δ*E*) of nonirradiated and irradiated Ag nanostructures with RCPL and various power densities of the CW laser. As irradiation was performed on the single sample, the time of irradiation accumulated with intervals of 2 min as power density increased to 5 W/cm^2^. After that, at power densities of 5 W/cm^2^, only accumulated time irradiation was increased according to following asterisks: * −23 min, ** −33 min, *** −60 min, **** −120 min. The incident wavelength is marked by vertical lines at 405 nm. HR-SEM images of (d) aged and (e) irradiated Ag NF. The insets depict the tilted HR-SEM images at the angle of 52 degrees.

Next, the aged Ag NFs with NPs formed on their surface were irradiated via a CW laser at 405 nm with varied power densities, irradiation time, and RCPL. The CD spectrum of the sample before irradiation in Figure 2a displays no optical activity, indicating that the NP ensemble is racemic. As irradiation power density increased up to 1.66 W/cm^2^, we can observe the appearance of a bisignate CD signal, peaking at wavelengths of 372 and 520 nm. Subsequent irradiation of the Ag NF at the same spot with a power density larger than 2.21 W/cm^2^ leads to a more complex shape of the CD spectrum with new CD bands at wavelengths of 401 and 447 nm. The increase in the negative CD signal at the excitation wavelength (405 nm) indicates a stronger absorption of probe RCPL by D-Ag NPs, corresponding to the same polarization of the source. With a further increase in the power density, the sign of CD at 405 nm changes to positive, which shifts the ratio of enantiomers in favor of L-Ag NPs that preferentially absorb LCPL. The maximum CD and the anisotropy factor (*g*-factor = 2 × (CD (mDeg))/(32,980 × Extinction)) at a wavelength of 401 nm are 26 mDeg and 0.2 × 10^–2^, respectively. Of particular importance is the intermediate range of 1.1 to 3.5 W/cm^2^. We found the relationship between the CD and power density at 405 nm (Figure S3), where, surprisingly, the CD signal remains stable, showing no significant change with increasing power. Such behavior may indicate the presence of multiple competitive processes influencing the NP reshaping and chirality as well. Note that the CD spectra and their time evolution are highly reproducible in repeated experiments with the same conditions and different film samples or different locations on the same substrate, and that they were stable for at least one month after irradiation when left at an ambient atmosphere.

Extinction of the pristine Ag NF (Figure 2b) is inhomogeneously broadened, which is obvious from the HR-SEM image in Figure 2d. Further analysis of the HR-SEM image shown in Figure 2e shows no background Ag film and increased Ag grains; hence, the extinction decrease in the long-wavelengths is associated with reshaping of the Ag NPs. Irradiation with RCPL not only increased the size of the Ag NPs but also significantly altered their shape, leading to the coalescence of several NPs into complex structures. Typically, this type of shape modification can be induced by laser heating, thermal annealing,^31,32^ ion beam irradiation,^33^ or inductively coupled plasma discharge,^34^ but in the present case, relatively low-power density has been used, which could not produce significant heating, as will be shown later.

In Figure 2b, we observe that the extinction in the short-wavelength range increases significantly with increasing irradiation power density, reaching a maximum close to the wavelength of 405 nm, which is typically characteristic of plasmon resonance in small Ag NPs less than 20 nm in size. Moreover, the inhomogeneously broadened extinction bands are determined not only by the plasmon resonance in the overgrown NPs but also by the absorption of the labyrinth-like film. Considering these findings, we assume that laser irradiation leads to thinning or even complete disappearance of the background film. The NPs become more rounded after irradiation, as seen in the SEM image in Figure 2e, taken at a 52-degree tilt. This fact is also confirmed by differential extinction spectra (Δ*E* = *E*_after_ – *E*_before_), where the growth of extinction is evident not only at 405 nm but also around 370 nm (Figure 2c). The emergence of a pronounced resonance at a wavelength of 370 nm, attributed to a quadrupolar resonance, implies that a transformation from a flat film to a 3D structure occurs under the influence of light.^25,35^ The quadrupole resonance mode in spherical Au and Ag NPs becomes more pronounced as the lower segment of the sphere is truncated to form a hemisphere-like structure.^36,37^

### Dynamics of Formation of Chiral Ag NF at Relatively Low Irradiation Power Densities

Figure 3a,b shows the evolution of the CD spectra of the Ag NF irradiated by 405 nm RCPL at 1.1 W/cm^2^ as a function of time estimated from Figure S4. Time dependence of CD (Figure 3c) and extinction (Figure 3d) at specific wavelengths was fitted by mono- and biexponential functions. We found that continued irradiation resulted in an increase in the absolute value of CD in the short-wavelength range, peaking at a wavelength of 405 nm. The increase in the CD signal at 405 nm, which is the irradiation wavelength, can be attributed to an increase in the proportion of D-Ag NPs that absorb RCPL more strongly than LCPL. The positive CD band spanning from about 450–500 nm toward the infrared region not only increases in magnitude but also undergoes a red-shift over the course of irradiation.

**Figure 3:**
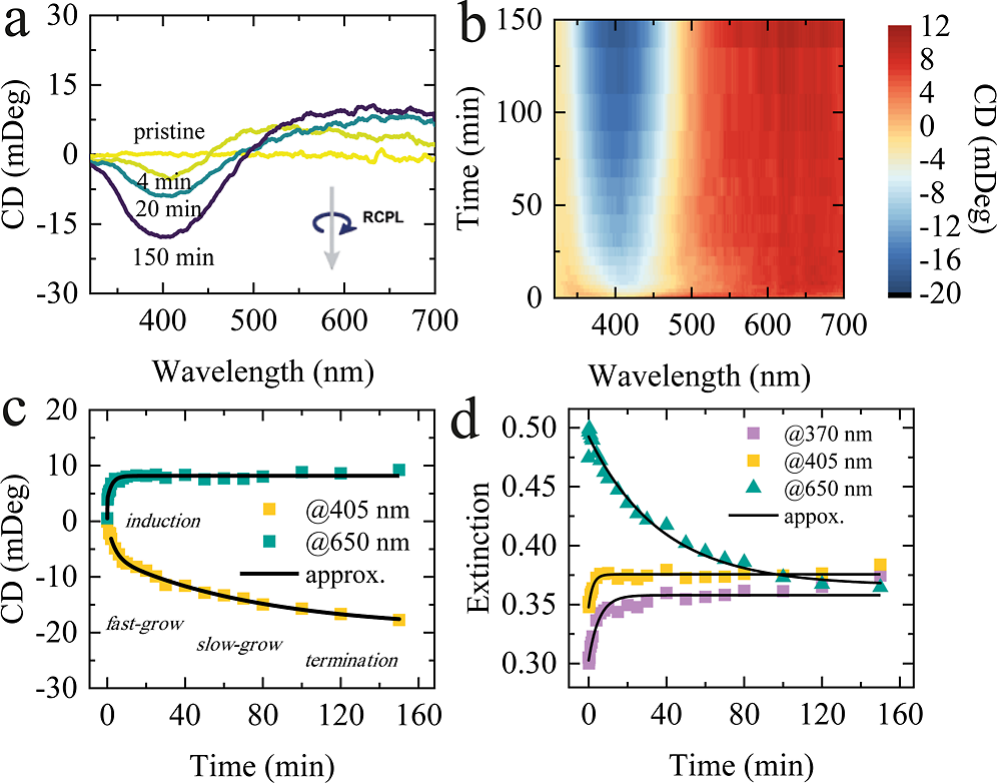
Time-dependent CD changes of Ag NF under RCPL: (a) CD spectra and (b) 2D map of CD as a function of 405 nm irradiation time. (c) CD growth kinetics monitored at 405 and 650 nm. (d) Extinction kinetics monitored at different wavelengths: 370 nm, 405 nm, and 650 nm. Power density was set as 1.1 W/cm^2^, where irradiation time was varied from 10 s to 150 min.

In order to analyze the rate of the reaction, we fitted the CD data at 405 nm by a biexponential function $A_{1}\cdot e^{-t/\tau_{1}}+A_{2}\cdot e^{-t/\tau_{2}}$ (Figure 3c), where an initial steep increase during irradiation with a decay time τ_1_ = 3.7 min was followed by a much slower growth rate, with a decay time τ_2_ = 76 min. For the extinction kinetics and CD signal at 650 nm, a monoexponential *A*·e^–*t*/τ^ curve was enough to fit the experimental data reasonably well. This might be explained by a significant increase in the number of NPs that are resonant with the incident light over the irradiation time, which allows for further shape transformations even at longer times. On the other hand, during irradiation at 650 nm, the value of CD changed rapidly over a short period of time and then saturated.

The reverse situation is observed for extinction kinetics, where one can see sharp changes occur over a short time span (2–3 min) in the short-wavelength region (370 and 405 nm), while the long-wavelength tail in the extinction spectra decreases exponentially over the decay time τ = 37 min. We hypothesize that the increase in the CD value over time is related to the growth and reshaping of D-Ag NPs driven by light-induced redistribution of silver. In aged samples, part of the silver is already present in an oxidized form, providing a reservoir of Ag^+^ ions. Under CPL irradiation, these ions are locally reduced and deposited onto resonant nanostructures at hot spots, leading to their preferential growth and, consequently, to an enhanced chiroptical response. Both extinction and CD kinetics suggest that the morphological changes in chiral NPs occur rapidly, while subsequent changes lead to only slight modifications, with CD growth exhibiting a prolonged and slowed response at 405 nm.

Additionally, we find out that, at relatively low-power densities (<1.1 W/cm^2^), the chiral transformation obeys the Bunsen–Roscoe reciprocity law, i.e., the CD response is proportional to the irradiation dose. In particular, Figure S5 and Table S1 show that when the same dose is applied using two different power densities and the corresponding irradiation times, the CD spectra are indistinguishable. These observations indicate that chiral NP growth is governed by a single-photon process.

It is evident that the shape of the bisignate CD bands is independent of irradiation time but largely determined by the irradiation power density. Notably, although the overall bisignate profile is preserved, the CD signal at the excitation wavelength (405 nm) undergoes a power-dependent sign inversion, being negative at relatively low-power densities (Figure 3a) and positive at high-power densities (Figure S6a,b). The change in the CD spectrum was also examined for the intermediate range of power density at 2.77 W/cm^2^, where we observed a sharp drop to −10 mDeg after 10 s of irradiation, after which the system became less sensitive to changes, as shown in Figure S6c,d. This behavior is attributed to the existence of a range of radiation power density values where the two effects: (i) light-induced growth and (ii) heat-driven reshaping, compensate for each other.

Next, we examined the effect of irradiation on freshly prepared Ag NF under RCPL by using a 405 nm laser. We applied a relatively high-power density of 5 W/cm^2^, the same as used for the aged sample, since no effect was observed at lower power densities. The negative CD value at 405 nm (Figure 4a) indicates that RCPL irradiation contributes to a slight increase in the amount of D-Ag NP. The low CD peak value of −3 mDeg is connected with reduced weak plasmonic effect (nonresonant film, Figure 4b) and insufficient oxidation. Yet, HR-SEM images (Figure 4c,d) show that irradiation leads to the formation of small NPs on top of the Ag NF.

**Figure 4:**
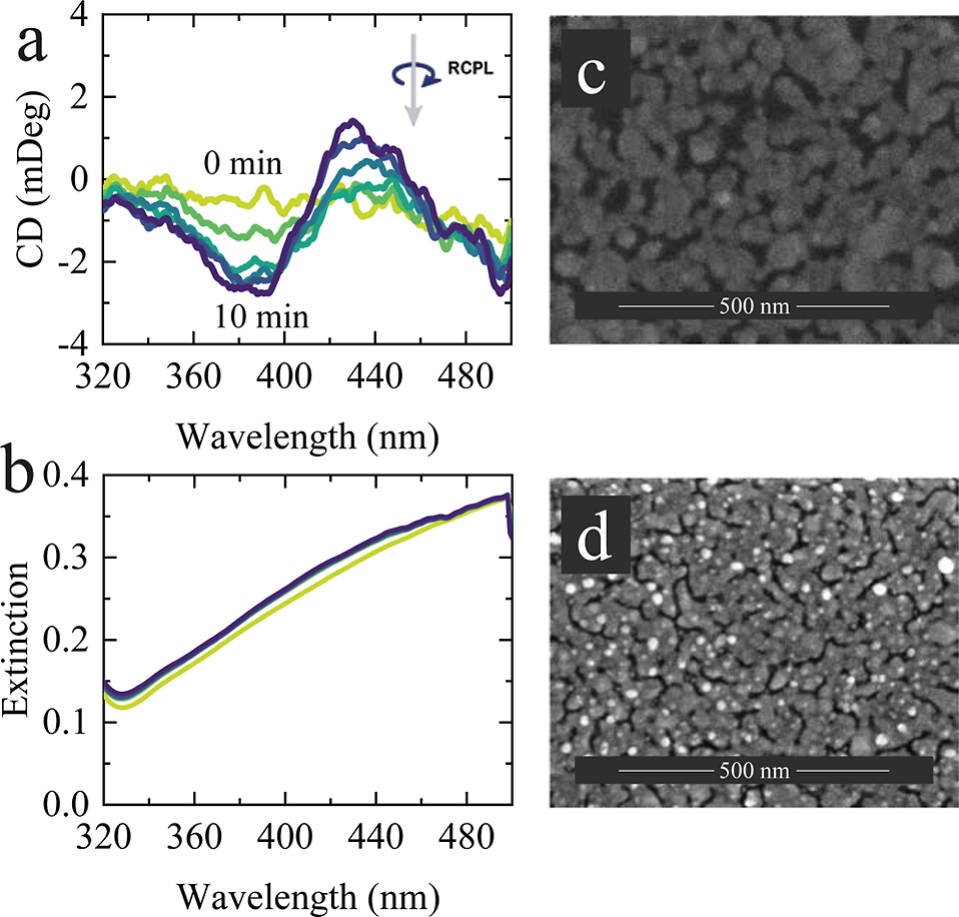
(a) CD and (b) extinction spectra of fresh Ag NF subjected to RCPL at the high-power density of 5 W/cm^2^. SEM images of fresh Ag NF (c) before and (d) after irradiation.

### The Role of a Silver Oxide Layer on Atom Diffusion

To better understand the different results on CPL-induced chirality between fresh and aged Ag NFs, we inspected the chemical state and elemental composition of the composite surface for two pristine substrates with Ag NPs (fresh and aged) by X-ray photoelectron spectroscopy (XPS) and analyzed them for the presence of an oxide layer (Figures 5, S16–S25, Tables S2 and S3). XPS/Auger data demonstrate that fresh and aged NFs are significantly different chemically. The XPS peaks of the aged sample, appearing at 368.5 and 374.5 eV (Figure 5a), can be attributed to a combination of Ag and Ag_2_O. The two peaks at 369.0 and 375.0 eV appearing in the fresh sample almost exclusively correspond to Ag^0^. The results of XPS and Auger spectra clearly prove the coexistence of a silver oxide layer in the aged substrate, while the fresh sample might have a negligible amount of oxide. Although, freshly prepared samples show a small sulfur contribution (∼160.7 eV) that disappears after aging, likely due to surface diffusion and progressive oxidation under ambient conditions (Supporting Information, Section S12). Thus, CPL-induced reshaping with a consequent imbalance of D-/L NPs or NP clusters is most effective in aged samples. While CD is observable on fresh substrates, it is notably weaker, which likely reflects the smaller concentration of mobile Ag^+^ ions present, with no significant changes to the film morphology (Figure 4c,d) when compared to the aged sample.

**Figure 5:**
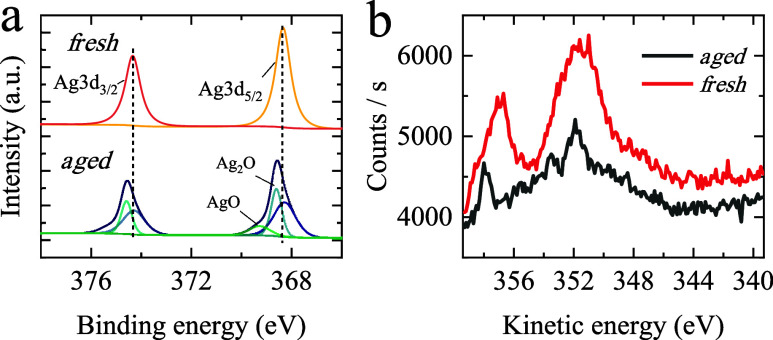
(a) XPS and (b) Auger spectra of fresh and aged NFs.

### Effect of the Handedness of Circularly Polarized Light

Figure 6 demonstrates that switching the excitation helicity from RCPL to LCPL reverses the sign of the induced CD across the spectrum, indicating the formation of enantiomeric chiral Ag NF governed by the incident CPL handedness. For instance, under a lower power density of 1.1 W/cm^2^ and a short irradiation time of 2 min (Figure 6a), the CD arising at the wavelength of the laser line (405 nm) was primarily due to increasing the number of Ag NPs matching the handedness of the laser polarization. When the power density and irradiation time were increased to 5 W/cm^2^ and 33 min (Figure 6b), the CD sign at 405 nm reversed, becoming opposite to the incident polarization with a more complex spectral line shape observed. Moreover, we also show that the CD spectrum of the Ag NF is invariant upon rotating and flipping of the sample, which in turn demonstrates that the Ag NFs possess genuine 3D chirality, rather than an effective planar chirality arising from anisotropic in-plane arrangements (Figure S7). Of course, irradiation of the Ag NF with linearly polarized light resulted in only a large linear dichroism (Figure S8), while no CD signal was observed. This confirms that the light-induced chirality is CPL-handedness sensitive and occurs specifically under chiral light.

**Figure 6:**
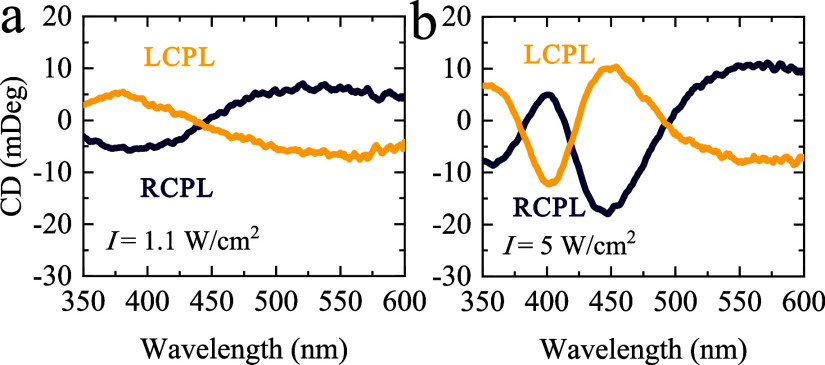
Representative demonstration of the formation of polarization-dependent chiral Ag NF prepared from the aged sample. CD spectra of chiral Ag NF fabricated via irradiation of initially achiral silver film by CPL with different handedness: LCPL (yellow curve) and RCPL (dark curves). The CD induction was done by the CW laser with a wavelength of 405 nm.

### Effect of the Wavelength of Circularly Polarized Light

Following the experiments with 405 nm irradiation, we also examined the impact of the CW laser irradiation at 532 nm. This wavelength is of particular interest, since it coincides with the maximum plasmon resonance of the NFs (Figure S9). Similar to irradiation at 405 nm, we observed an alternating polarity CD signal with an increasing peak intensity at 532 nm (Figure 7), indicating the dominant presence of L-Ag NPs, opposite of the polarization of the incident light. The increased CD magnitude observed when the irradiation wavelength aligns with the plasmon resonance maximum is probably due to the increased number of NPs participating in the light-induced reshaping process, as well as the larger absorption cross-section of the excited NPs compared to those that absorb 405 nm light. Since the irradiation was applied sequentially to the same sample at different time intervals, it is reasonable to expect that the observed processes exhibit an accumulative nature and, under certain conditions, will reach saturation, as shown in the inset of Figure 7a. The magnitude of the CD peak after irradiation at 532 nm is more than twice the one from 405 nm irradiation under identical power density conditions. The highest CD and *g*-factor values observed were 168 mDeg and 1.2 × 10^–2^, respectively. In contrast to the 405 nm CW irradiation, for which the CD sign at the laser wavelength reverses with increasing power density, we found that under 532 nm irradiation, the CD sign at the laser wavelength remains unchanged over the entire range of power densities studied (Figure S10). SEM images postirradiation show the agglomeration of several individual NPs, likely associated with Ostwald ripening. Specifically, the ripening process implies dissolving small NPs and redistributing Ag atoms to larger ones.

**Figure 7:**
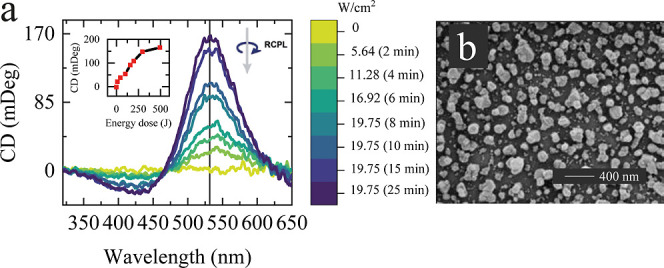
(a) CD spectra of as-deposited and irradiated Ag NFs with various power densities of the RCPL CW laser at 532 nm. Irradiation was done on the single sample, with the NF exposed to accumulated irradiation intervals of 2 min with increasing power density. The inset shows the CD magnitude at 532 nm as a function of energy dose. (b) The HR-SEM image of an irradiated aged Ag NF.

### The Photophysical Mechanisms of the Chiral Induction in a Random Array of NPs

To elucidate the mechanism of CD induction, we first examined morphological changes in Ag NFs before and after irradiation by acquiring SEM images from the same location while systematically varying the power density (0.5–5 W/cm^2^), the exposure time (2 and 20 min), and the laser wavelength (405 and 532 nm). The SEM images in Figure 8 demonstrate that at low laser power densities, NPs nucleate and grow both on pre-existing seeds and in previously unoccupied regions of the substrate. When the power density of the 405 nm irradiation is increased to 5 W/cm^2^, the initial NPs progressively transform into larger, nearly spherical NPs, which grow at the expense of smaller ones. In contrast, under 532 nm irradiation at the same power density, only a general dissolution of NPs is observed. This coarsening behavior is consistent with a model of light-induced Ostwald ripening.

**Figure 8:**
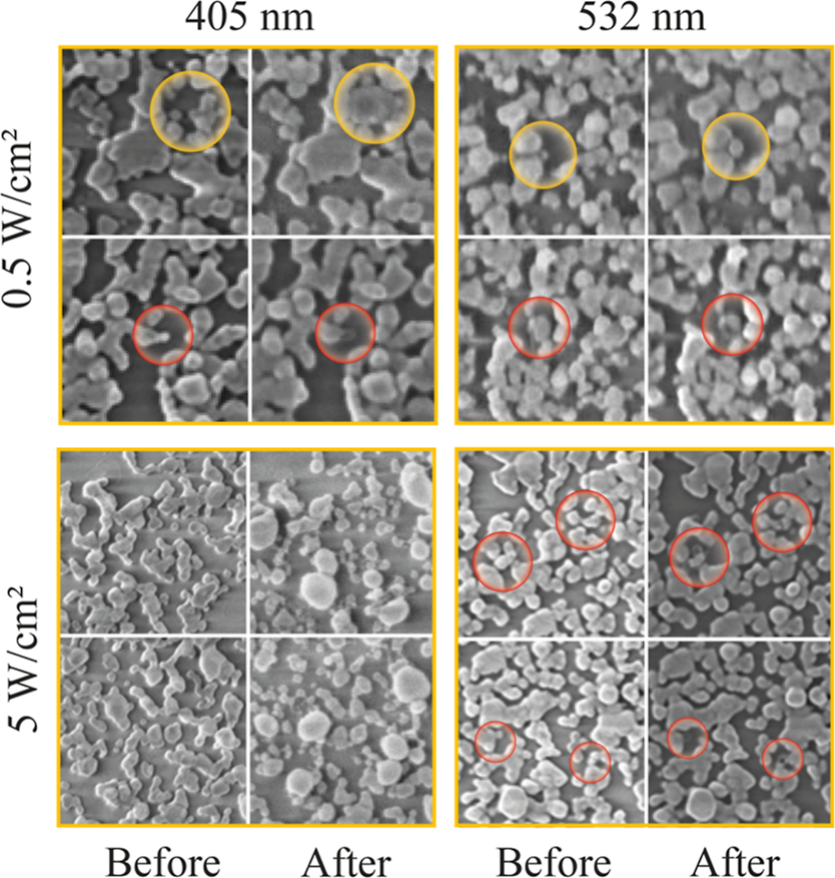
SEM images of Ag NFs before and after CPL irradiation with various laser sources demonstrating dissolution, nucleation, and fragmentation.

Based on the SEM results, the power dependence of the peak CD is the result of two competing processes: silver dissolution and (re)­deposition. In the case of RCPL illumination with a power density of less than 1.1 W/cm^2^ and a wavelength of 405 nm, the increase in CD intensity is due to the preferential growth of D-NPs, while at relatively high-power densities, the opposite effect occurs, associated with the reduction of resonant D-NPs. The proposed photophysical mechanism of chiral optical imprinting in a random Ag NP array is summarized in Figure S12. Under CPL illumination, isolated symmetric NPs do not develop any chiral growth bias, whereas symmetric dimers and more complex clusters experience an asymmetric distribution of plasmonic “hot spots” that may drive handedness-selective growth or dissolution. As a result, initially achiral NPs (dimers or trimers) are progressively transformed into chiral structures with a nonzero chiral growth coefficient (*C*), while pre-existing asymmetric Γ-type trimers have their initial handedness further amplified (or diminished, in case of opposite CPL). Therefore, we believe that the growth of chiral NPs in our system is governed by plasmon-induced redox processes, in which Ag^+^ ions or Ag^0^ atoms interact with hot carriers (HEs—hot electrons and HHs—hot holes) generated at specific locations near the NP surface. Also, we assume that due to the presence of a semiconductor shell made of Ag_2_O, HEs with sufficient energy can be injected into the conduction band of Ag_2_O, overcoming the Schottky barrier formed at the Ag/Ag_2_O interface.^38^ The presence of an oxide layer may enhance the efficiency of HE transfer from the Ag surface and facilitate the reduction of Ag^+^ to metallic silver (Ag^0^). Namely, we assume that Ag^+^ ions diffuse at the surface to reach the plasmonic hot spots and get deposited to subsequently form chiral Ag NF. Also, HHs might be producing more Ag^+^ during illumination in different places if the HHs have different mobilities and/or decay rates compared with HE. In turn, high mobility of Ag^+^ ions can be explained by the presence of moisture in the surrounding environment, consequently forming a thin layer (1–2 nm thick) of adsorbed water.^39^ This thin layer of water can dissolve Ag^+^ ions, allowing them to diffuse across the surface of the glass substrate and Ag NF.

To support our assumption about silver growth mediated by HEs in Figure 9, we provide numerical simulations. We used the following notation: CD in transmission was defined as CD_*T*_ = *T*_LCPL_ – *T*_RCPL_. Correspondingly, since the extinction in the model is given by Ext = 1 – *T*, the extinction CD satisfies CD_*T*_ = −CD_Ext_. For the CD of the HE-generation rate, we have, respectively: CD_Rate_ = Rate_LCPL_ – Rate_RCPL_, where Rate_LCPL_ and Rate_RCPL_ are the total (i.e., integrated over surface) rates of HE generation. The equations to compute the rates via COMSOL are given in the Methods section. We first consider a single hemispherical NP, which is intrinsically achiral (Figure S13). As expected, all CD responses vanish in this case since Rate_LCPL_ = Rate_RCPL_ = Rate_hot‑e,tot_ (Figure S13). For the asymmetric dimer (Figure S14), the physical situation is fundamentally different. Although the overall CD remains zero, the local optical response becomes strongly chiral. Under circularly polarized irradiation, the system exhibits a spatially asymmetric ${\left|E\right|}^{2}$ distribution (Figure 9a) and a pronounced difference between the local HE generation rates for L- and R-CPL, as shown in Figure 9b. Full theoretical formalism is provided in the Methods section. The local CD functions of local rates of HE generation in Figure 9b are denoted and computed as rate_CD_(*x*) = rate_LCPL_(*x*) – rate_RCPL_(*x*), where rate_LCPL_(*x*) and rate_RCPL_(*x*) are the local, position-dependent rates of HE generation under for L- and R-CPL; the *g*-factor of the chiral HE generation maps is denoted as *g*_HE_ and computed according to the formalism in the Methods section. The *g*-factor spectrum shows the appearance of 2D chirality in the local HE rates. As shown in Figure 9b, this effect leads to the formation of spatially localized chiral hot spots of HE generation, which provide the microscopic origin of the directional growth processes illustrated in Figure S12. These chiral hot spots ultimately drive the emergence of the quasi-2D chiral morphology observed experimentally in the Ag NF.

**Figure 9:**
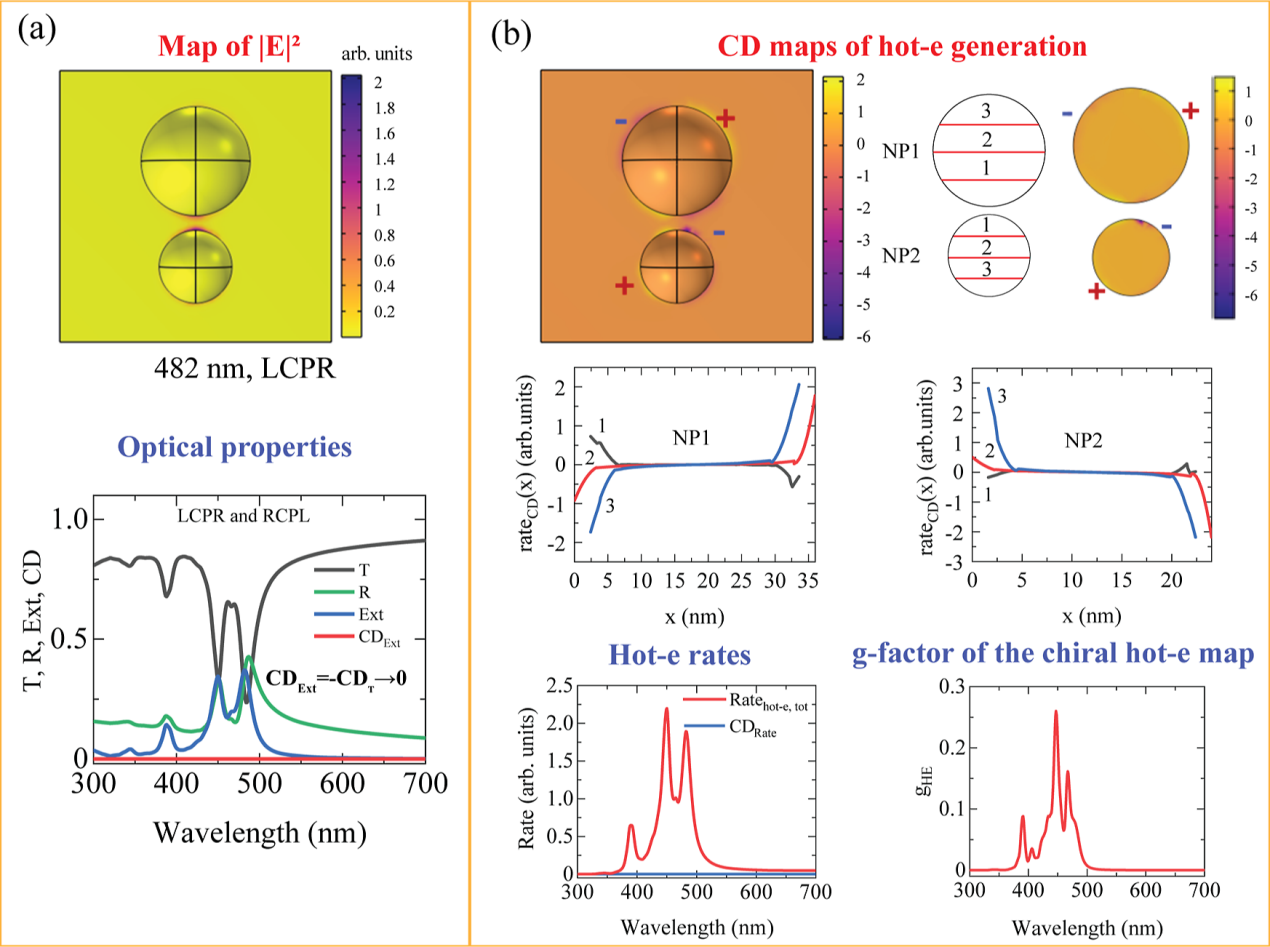
(a) Calculated *E*-field maps at 482 nm under LCPL and optical spectra, including transmission (*T*), reflection (*R*), and extinction (Ext) for two unequal hemispherical Ag NPs on a glass substrate. (b) Calculated CD local maps of the HE generation rate (shown for the upper and lower NP surfaces), corresponding CD spatial profiles along lines 1, 2, and 3, and the HE generation rate and *g*-factor spectra.

The second mechanism, which becomes dominant at higher irradiation power densities, is most likely governed by photothermal reshaping and coarsening of the Ag NF. Under these conditions, plasmon-induced heating can cause the NPs to partially melt and relax into more compact, nearly spherical shapes and, in some cases, to coalesce with nearby particles. In addition, smaller NPs with higher surface energy may preferentially dissolve or be consumed under illumination, while larger ones grow at their expense, in line with an Ostwald ripening-type process. Together, these thermally driven reshaping, coalescence, and size-dependent dissolution pathways lead to the preferential degradation of the initially defined D-Ag NPs by RCPL and the formation of coarsened, largely nonresonant nanostructures. To assess macroscopic photothermal heating of the Ag NF, we monitored the sample temperature during laser irradiation using an infrared thermal camera (Figure S11). For low incident power, no noticeable temperature change was detected within the spatial resolution of the camera. At high power density, however, the maximum temperature in the laser-irradiated region increased from ≈23 °C (laser off) to ≈30 °C (laser on), i.e., by about 7 °C. Although this macroscopic temperature rise is relatively modest, the IR images confirm that the film efficiently absorbs the incident light and undergoes photothermal heating. We note that the thermal camera provides an averaged temperature over the laser spot and the substrate surface. Furthermore, thermal reshaping was previously observed in similar films through heating to >70 °C.^32^ To verify the existence of plasmon-induced Ag dissolution, we synthesized colloidal Ag NPs and exposed them to polarized light at 405 and 530 nm from LED sources (Figure S15). After exposure, the solutions were analyzed using inductively coupled plasma mass spectrometry (ICP–MS) to quantify the concentration of Ag^+^ released into the solution (see Table 1). Under low-power LED illumination (0.83 W/cm^2^) at 405 or 530 nm, the concentration of free Ag^+^ decreased, indicating the preferential reduction of silver ions by hot electrons. At a higher irradiation power (2.84 W/cm^2^), the response became wavelength-dependent. Under 530 nm illumination, the Ag^+^ concentration increased, suggesting dominant dissolution of Ag from the NPs, whereas at 405 nm, it still decreased, although it was less pronounced than at low power. We assume that at high power, pronounced morphological changes were observed due to Ag dissolution, followed by redeposition; however, at 405 nm, redeposition dominated over dissolution, resulting in a net decrease in free Ag^+^.

**Table 1:** ICP–MS Results

Irradiation wavelength	Irradiation conditions	Ag^+^ conc. [ng/mL]	Ag^+^ conc. [μM]
None	control	455.3	4.22
530 nm	0.83 W/cm^2^	386.7	3.58
	2.84 W/cm^2^	705.3	6.54
405 nm	0.83 W/cm^2^	315.4	2.92
	2.84 W/cm^2^	336.7	3.12

## Conclusions

In conclusion, we have demonstrated a facile light-based approach to imprint chirality in initially achiral Ag NF obtained by physical vapor deposition on a dielectric substrate. Under continuous-wave CPL illumination, chirality emerges as localized, asymmetric plasmon-induced metal redeposition and reshaping which disrupt the balance between left- and right-absorbing NP “enantiomers”. Chirality induction in this process is determined by two laser regimes: at low power densities, the dominant mechanism is the generation of hot carriers, which drive redox processes and atomic diffusion, while at high power densities, heating plays a dominant role, becoming the main factor in structural transformation. The resulting films exhibit sizable *g*-factors, despite the absence of any chiral molecules or lithographic patterning. Our results show that CPL-driven laser reshaping of structurally disordered metal films provides a versatile platform for fabricating large-area chiral plasmonic substrates and suggest new opportunities for scalable chiroptical metasurfaces, polarization-sensitive components, and enantioselective sensing interfaces. One possible chiral molecular sensing mode, which is enabled by our method, would be imprinting adjacent right- and left-handed areas on the same substrate through alternating LCPL and RCPL irradiation and detection of the differential optical response from those two areas to adsorption of chiral molecular samples.

## Methods

### Fabrication of Silver Films

The microscope glass substrates were cleaned with ethanol and deionized water. Then, thin films were thermally evaporated on the substrates via physical vapor deposition of pure silver (99.99%) in a vacuum chamber PVD-75 (Kurt J. Lesker) with a base pressure of 5 × 10^–7^ Torr. The films consisted of self-organized nanostructures formed according to a Volmer–Weber growth mechanism. The equivalent film thickness was 12 nm, at a deposition rate of 0.5 Å/s, and the substrate was maintained at room temperature. The thicknesses of the silver films was monitored using a quartz crystal microbalance sensor.

### Spectral and Structural Property Measurements

The extinction and CD spectra in the wavelength range of 320–700 nm were measured using UV–vis spectrometers SF-56 (LOMO, Russia) and JASCO-1500 (JASCO, Japan) CD spectrometer, respectively. The CD measurement data were generally reported in ellipticity (Θ) units. Ellipticity is expressed as the product of 32,980 and the difference in extinction of the solid sample illuminated with LCPL and RCPL (Θ = 32,980 × ΔExtinction_LCPL–RCPL_). The morphology characterizations of the self-organized Ag NF were performed by using SEM analysis with a Helios G4 instrument. XPS measurements of fresh and aged samples were performed using an ESCALAB QXi (Thermo Scientific, USA). The binding energies were corrected using the 1 s peak of adventitious carbon fixed at 284.8 eV. Auger parameter was taken as Ag 3d_5/2_ + M_5_N_4_5N

### Laser Irradiation with Circularly Polarized Light

The silver NFs were irradiated using CW lasers at wavelengths of 405 and 532 nm. The power density of irradiation varied from 0 to 19.75 W/cm^2^. The beams’ spot sizes were estimated to be 5 and 1.2 mm for sources at λ = 405 nm and λ = 532 nm, respectively. The linearly polarized light from the CW lasers was converted to circularly polarized light by rotation of the zero-order quarter waveplate at +45° and −45° in respect to the polarization axis of the laser. The samples were irradiated at the same spot, accumulating the irradiation effect over all irradiation intervals. Following each irradiation step, the extinction and CD spectra were recorded.

### Synthesis of Colloidal Ag Nanospheres (Ag NPs)

To synthesize colloidal silver nanospheres (Ag NPs), 625 μL of 4 mM AgNO_3_ solution was added to 9.325 mL of ultrapure H_2_O and mildly stirred for 30 s. Then, under vigorous tiring, 50 μL of NaBH_4_ 0.3 M was quickly injected into the vial. A yellowish-brown color was immediately observed further developing for about 2 min. The reaction was stirred for another 15 min. The resulting Ag NPs were stored in a dark place and used within 72 h.

### Inductively Coupled Plasma Mass Spectrometry

The Ag NP solutions after illumination under different conditions were centrifuged at 15,000 rpm for 4 min to precipitate out the NPs, following which the supernatants were collected for evaluation. The concentrations of Ag^+^ ions in these solutions were determined by ICP–MS (Agilent model 7800) to monitor the Ag^+^ dissolution and deposition during irradiation. This was accomplished by heating a mixture of 0.5 mL of supernatant with 1.5 mL of 32% hydrochloric acid and 0.5 mL of 67% nitric acid for 1 h at 75 °C. The mixture was further diluted with distilled water to a final volume of 50 mL and then injected into the ICP–MS instrument. A calibration curve for the ICP–MS was prepared from a standard solution of the Ag ions and was used for quantitative concentration determination. A control experiment, in which the Ag NPs were directly centrifuged without illumination, showed a silver ion concentration of 4.22 μM, probably originating from unreacted silver ions in the reaction mixture.

### Numerical Calculations and Hot Electron Generation Formalism

Electromagnetic simulations were performed to model the optical properties and HE generation rates of hemispherical Ag NPs. We used a commercial finite-elements method software (COMSOL Multiphysics software, with the RF module). We used NPs of 12 and 18 diameters. The dielectric function of Ag was taken from the data set of Johnson and Christy,^40^ while the permittivity of the glass substrate was set to ε_sub_ = 2.25. We have recently developed a HE generation formalism that is suitable for NPs in solution and on substrates (see refs 14, 15, and 41 ). In general, it has been shown that HE generation happens at the NP surface, driving local growth, etching, and other chemical transformations. We can simulate either the far-field spectra or local responses, such as surface maps of the HE generation rates, for example. The rates are computed for the energetic electrons (*E*_F_ + *ℏω* > *E* > *E*_F_), which are able to efficiently induce reactions at the surface.

1 $${Rate}_{\mathrm{HE,tot}}=\frac{1}{4}\times \frac{2}{\pi^{2}}\times \frac{e^{2}E_{F}^{2}}{\hbar }\frac{\left(\hbar \omega -\Delta E_{bar}\right)}{{\left(\hbar \omega \right)}^{4}}\int_{S_{NP}}{\left|E_{\omega ,normal}\left(\theta ,\varphi \right)\right|}^{2}\;ds$$

where *E*_ω,normal_(θ, φ) is the normal component of the electric field at the surface of the NP; *E*_F_ = 5 eV is the Fermi energy for silver. The integral is taken over the NP’s surface. Accordingly, the local HE generation maps are calculated as

2 $${Rate}_{HE}\left(r\right)=\frac{1}{4}\times \frac{2}{\pi^{2}}\times \frac{e^{2}E_{F}^{2}}{\hbar }\frac{1}{{\left(\hbar \omega \right)}^{3}}{\left|E_{\omega ,normal}\left(\theta ,\varphi \right)\right|}^{2}$$

The local CD map and the *g*-factor for the HE generation rate excited by LCPL and RCPL are calculated according to eqs 3 and 4:

3 $${Rate}_{CD}\left(r\right)={Rate}_{LCPL}\left(r\right)-{Rate}_{RCPL}\left(r\right)$$

$$g_{HE}\left(\lambda \right)=\frac{\int_{\text{All}\;Surface}\left|{Rate}_{LCPL}\left(r\right)-{Rate}_{RCPL}\left(r\right)\right|\;ds}{\left(\int_{\text{All}\;Surface}{Rate}_{LCPL}\left(r\right)\;ds+\int_{\text{All}\;Surface}{Rate}_{RCPL}\left(r\right)\;ds\right)/2}$$ 4

Here, Rate_LCPL_(*r*), Rate_RCPL_(*r*), and Rate_CD_(*r*) are the corresponding local rates. The spectrum *g*_HE_(λ) represents the global *g*-factor of HE generation over all surfaces of a given NP and includes the corresponding integrals.

## Supplementary Material



## Notes

SEM characterization of aged nanostructured Ag films and NP size distribution; CD spectra versus power density and dose-dependent comparisons; extinction/differential extinction spectra before and after CW CPL irradiation at 405 and 532 nm (including RCPL kinetics); orientation-dependent measurements; thermal-camera monitoring; COMSOL model parameters; and characterization of colloidal Ag NPs used for ICP–MS (PDF)
