# Supplementary material for: Chiral Transformation of a Nanostructured Silver Film by Illumination with Circularly Polarized Light
Source: ACS Nano. 2026 Mar 4;20(11):9516–25. doi: 10.1021/acsnano.6c00256 (PMC13019665; doi:10.1021/acsnano.6c00256)
Supplement: Supplementary file 1 [file nn6c00256_si_001.pdf]

Supplementary Materials

Chiral Transformation of a Nanostructured Silver Film by Illumination with Circularly Polarized Light

Daler R. Dadadzhanov*,[†,‡], Nikita S. Petrov, Igor A. Gladskikh,[‡]
Daniel Feferman,[†] Nikita A. Toropov,[‡,¶] Leilei Gu,[§] Peng Yu,[‖] Zhiming Wang,[‖]
Tigran A. Vartanyan,*,[‡] Alexander O. Govorov[§] and Gil Markovich*,[†]

[†]*Raymond and Beverly Sackler Faculty of Exact Sciences, School of Chemistry, Tel Aviv University, Tel Aviv 6997801, Israel*
[‡]*International Research and Education Centre for Physics of Nanostructures, ITMO University, St. Petersburg, 197101, Russia*
[¶]*University of Southampton, Southampton, SO17 1BJ, United Kingdom*
[§]*Department of Physics and Astronomy, Nanoscale and Quantum Phenomena Institute, Ohio University, Athens, OH, 45701 USA*
[‖]*Institute of Fundamental and Frontier Sciences, University of Electronic Science and Technology of China, Chengdu, 610054, China*

E-mail: daler.dadadzhanov@gmail.com, tigran.vartanyan@mail.ru, gilmar@tauex.tau.ac.il

1. *Morphology of nanostructured Ag film before and after aging*

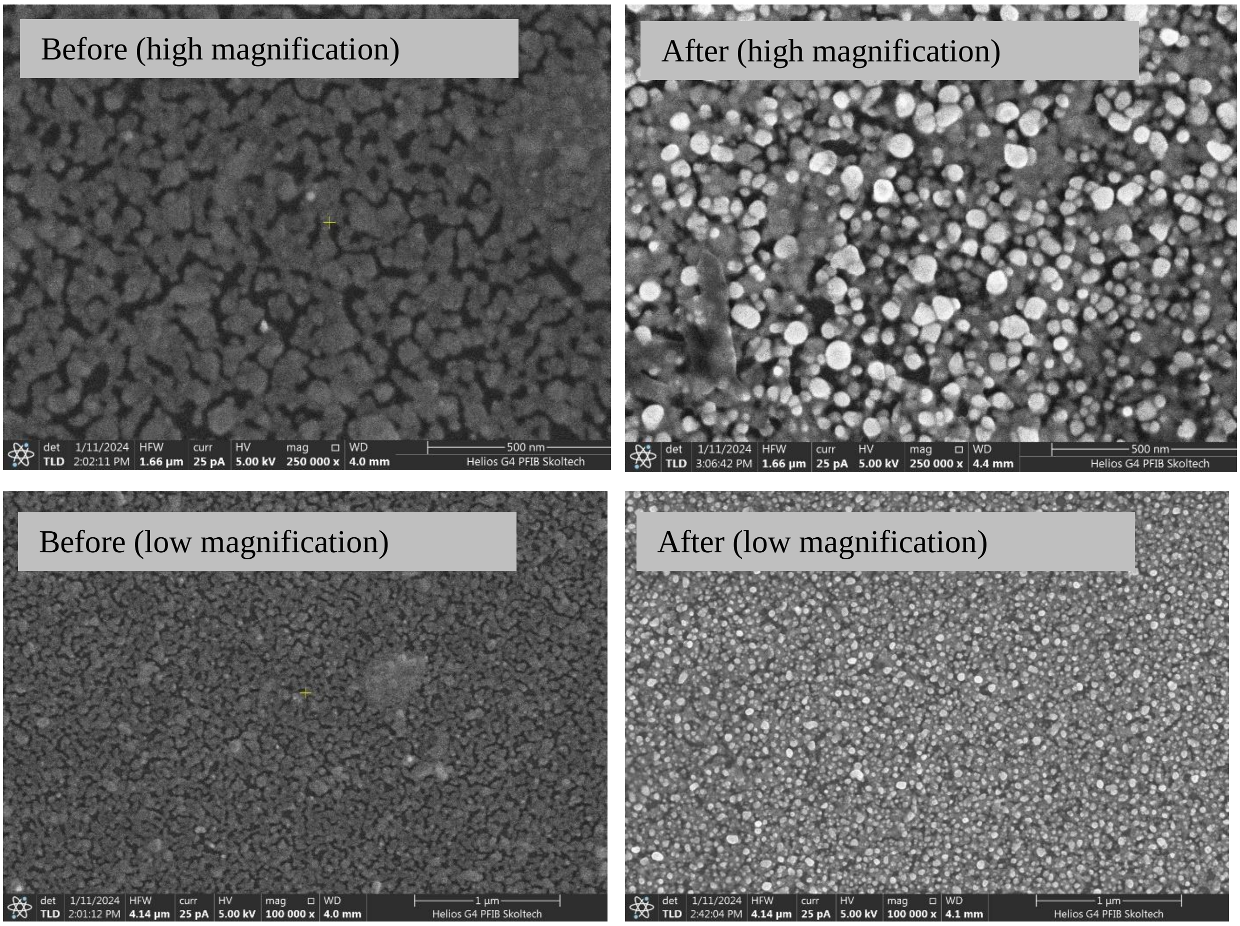

Figure S1. HR-SEM images of the Ag NF with the equivalent thickness of 12 nm before and after aging during several weeks in ambient conditions at room temperature: (top) – high and (bottom) low magnifications.

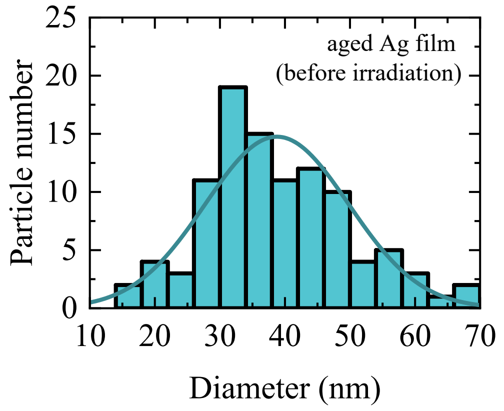

Figure S2. The size distribution of NPs on aged Ag NF before irradiation.

2. Effect of various power density with RCPL irradiation at ***405 nm***

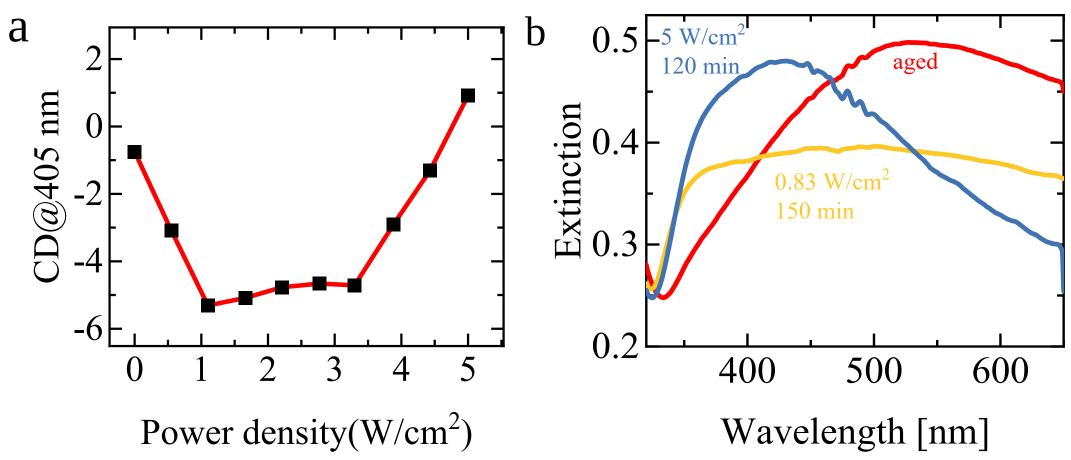

Figure S3. (a) The dependence of CD at wavelength of laser irradiation (405 nm) as function of power density. This plot was estimated from Figure 2a. (b) Extinction spectra of Ag NF before and after irradiation by CW laser with RCPL with various power density.

3. Time-dependent measurements of extinction and differential extinction for Ag NF

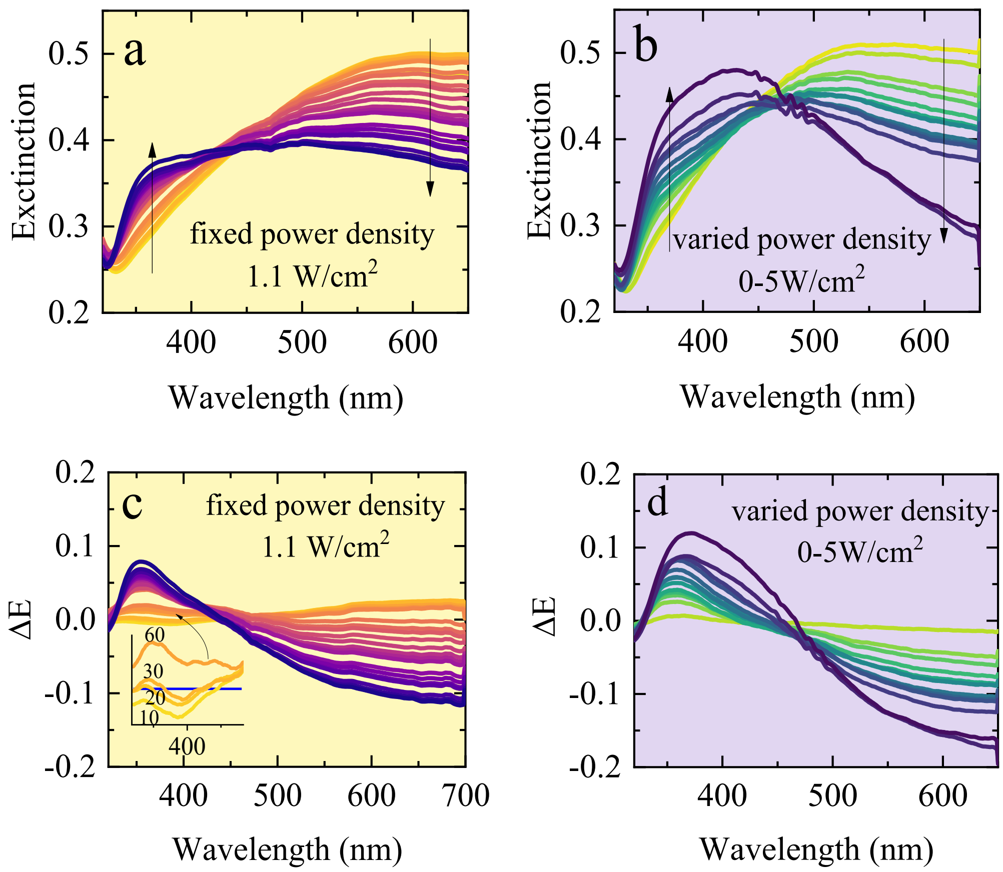

Figure S4. Extinction and differential extinction spectra of aged Ag NF. In case of (a) and (c) power density was fixed as 1.1 W/cm^2, while for (b) and (d) the power density varied in the range of 0.55-5 W/cm^2.

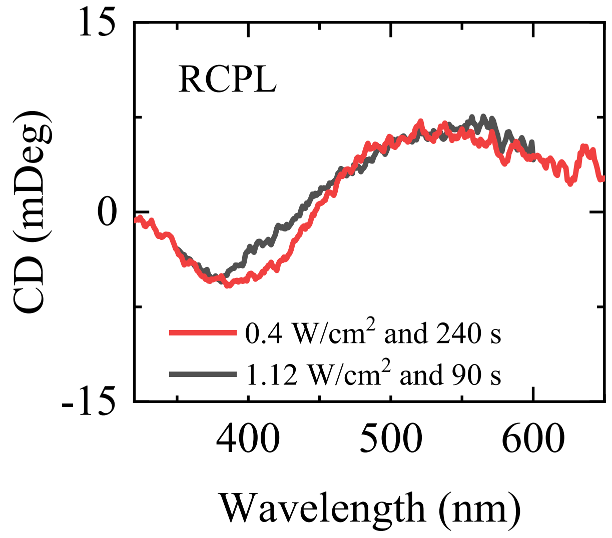

Figure S5. The comparison of CD spectra of nanostructured Ag NF after irradiation with different intensity and different duration but the same irradiation dose.

Table S1. Verification of one-photon absorption mechanism at low power densities.

Sample	Power (mW)	Power density (W/cm^2)	Exposure time (s)	Irradiation dose (mJ/cm^2)	Photon count (×10^{19}) 1/cm^2
**Ag_1**	80	0.4	240 s	0.27	3.91
**Ag_2**	220	1.12	90 s	0.28	4.03

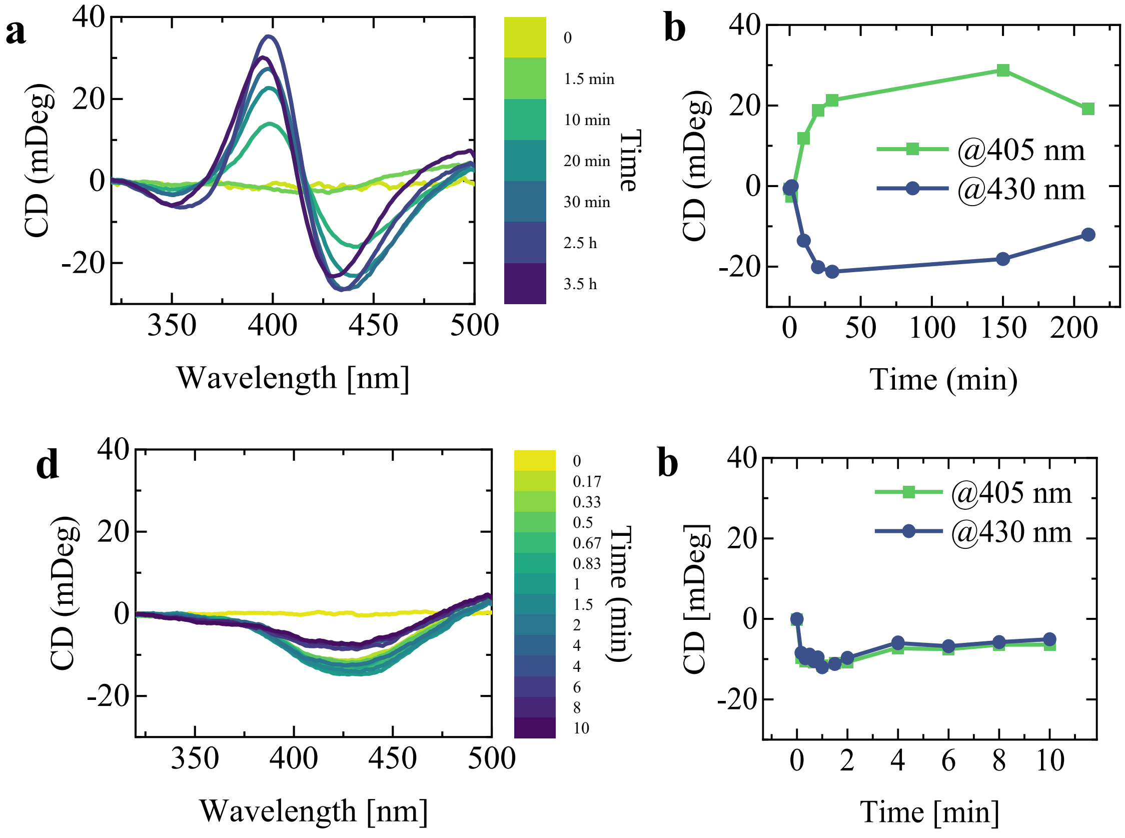

Figure S6. Irradiation with RCPL at aged Ag NF with different power density and under RCPL at 405 nm: (a, b) – 5 W/cm^2 and (c, d) – 2.77 W/cm^2. (a, c) CD spectra of nanostructured Ag NF and (b, d) CD kinetics at certain wavelengths 405 nm and 430 nm.

4. *Evidence that the observed circular dichroism is due to 3D chiral nanoparticles rather than to 2D anisotropic nanostructures*

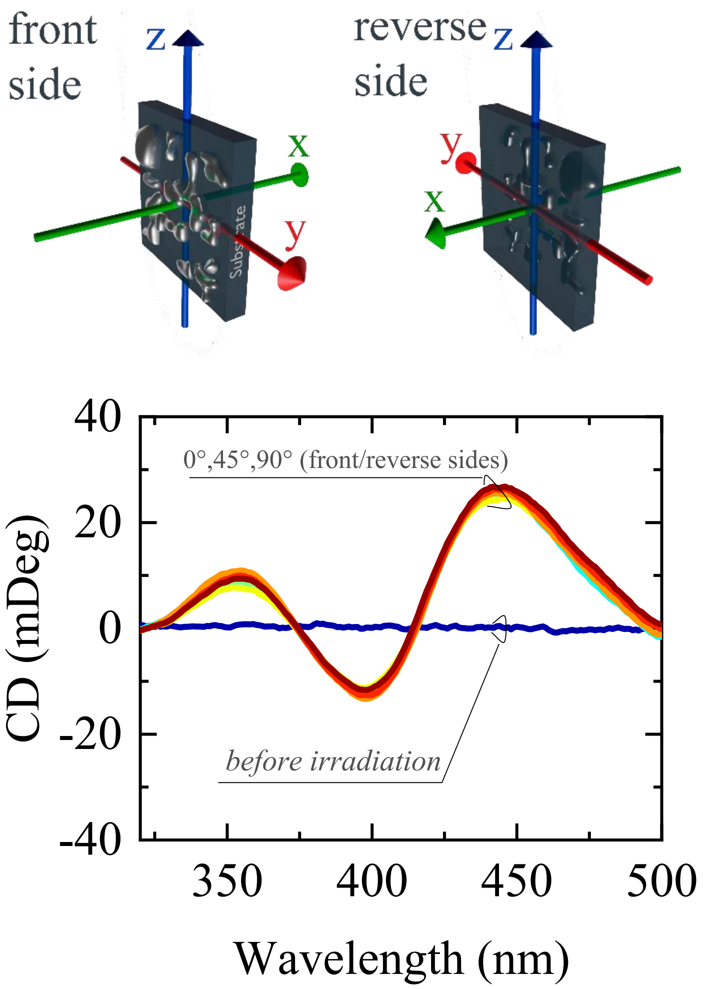

Figure S7. The scheme of sample measured in different orientation and propagation direction of probe beam and corresponding (b) CD spectra of silver nanostructures. The sample was irradiated by RCPL at the power density of 5.26 W/cm^2 for 5 min.

5. *Irradiation with linearly polarized light*

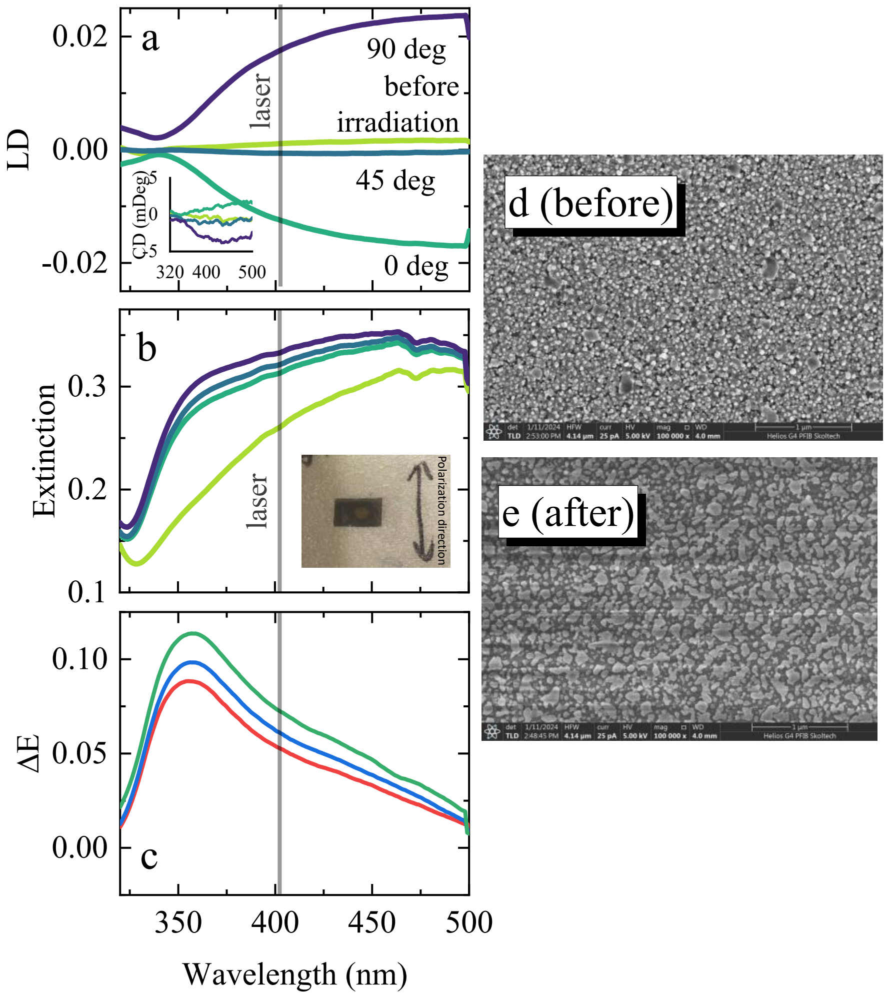

Figure S8. (a) Linear dichroism (LD), extinction, and differential spectra of Ag nanoflowers (Ag NFs) measured before and after illumination with linearly polarized laser radiation. The LD spectra were recorded with the probe beam polarization oriented either parallel (0°) or perpendicular (90°) to the polarization direction of the irradiation laser. (d, e) HR-SEM images of the same Ag NFs prior to and following laser irradiation. The laser power density and exposure time were 5 W/cm² and 5 min, respectively.

6. Extinction spectra of aged Ag NSs with RCPL irradiation at 532 nm

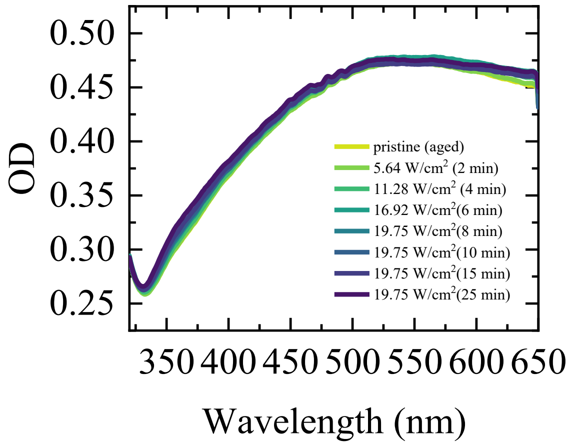

Figure S7. Extinction spectra of as-deposited (yellow curves) and irradiated Ag NF (other curves) with various power density of CW laser at 532 nm under RCPL.

7. Effect of various power density with RCPL irradiation at 532 nm

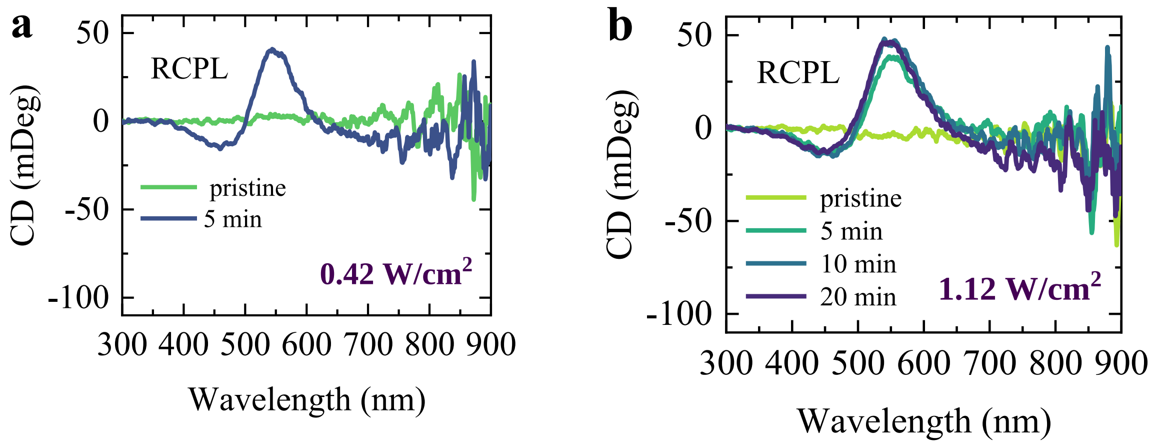

Figure S8. Irradiation with RCPL at aged nanostructured Ag film with different power density and under RCPL at 532 nm: (a) – 0.42 W/cm^2 and (b) – 1.12 W/cm^2.

8. *Images from thermal camera during laser irradiation of Ag NSs*

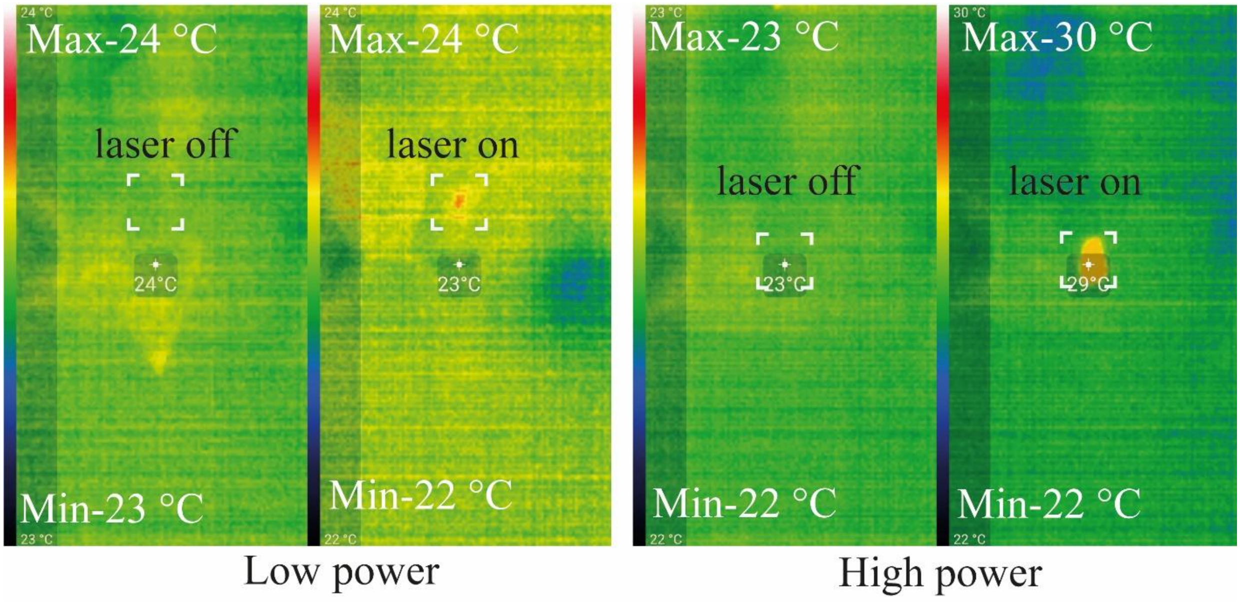

Figure S11. The images from thermal camera before and during laser irradiation with 405 nm laser. Low power is 1.1 W/cm^2, while high power is 1.1 W/cm^2.

9. *Mechanistic view of chiral optical imprinting*

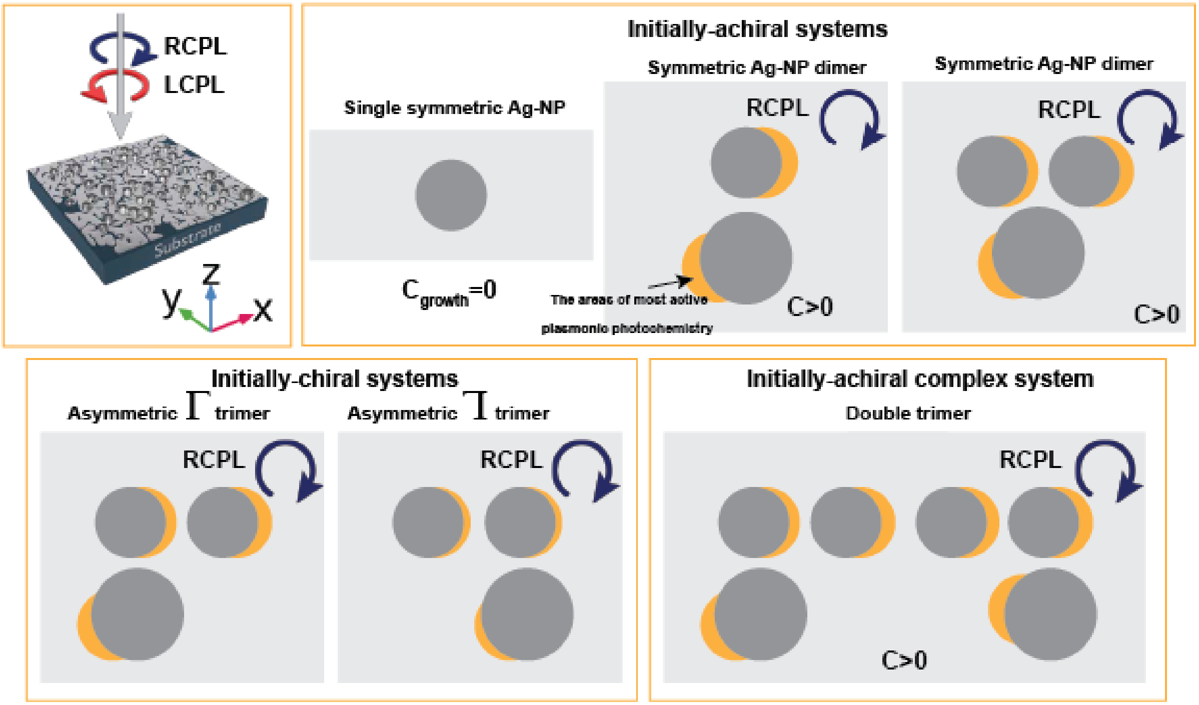

Figure S12. The photophysical mechanism of the chiral optical imprinting in a random array under CPL.

10. Numerical simulations

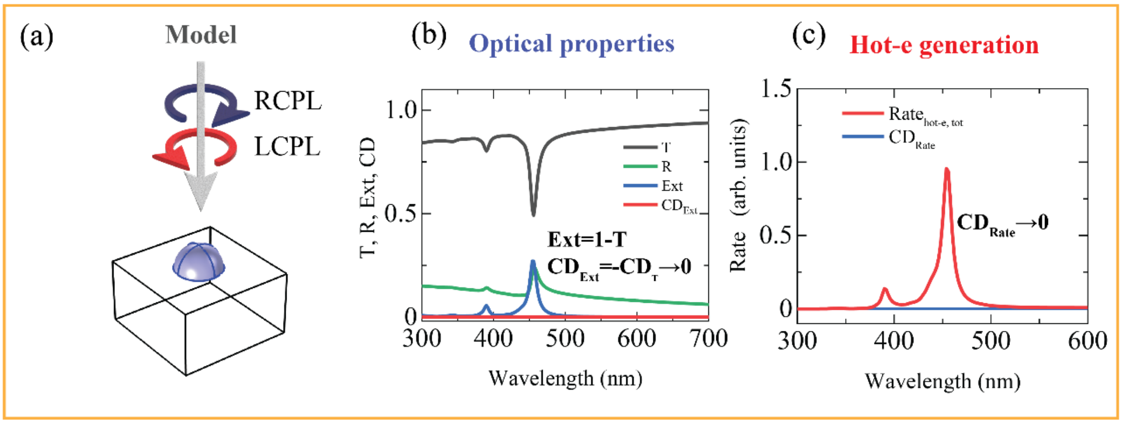

Figure S13. (a) Model used for calculation and optical spectra for a single Ag NP on a glass substrate, including (b) transmission (T), reflection (R), extinction (Ext), and (c) hot-electron (HE) generation rates spectra.

Single-NP model:

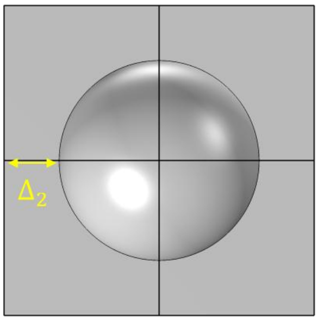

COMSOL, periodic BC
Periods: px, py

R1 = 18nm
Δ_2= 10[nm]
p_x = 2(R1) + 2Δ_2= 56[nm]
p_y = 2(R1) + 2Δ_2= 56[nm]

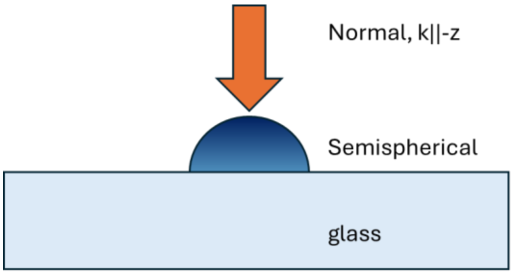

2-NP model:

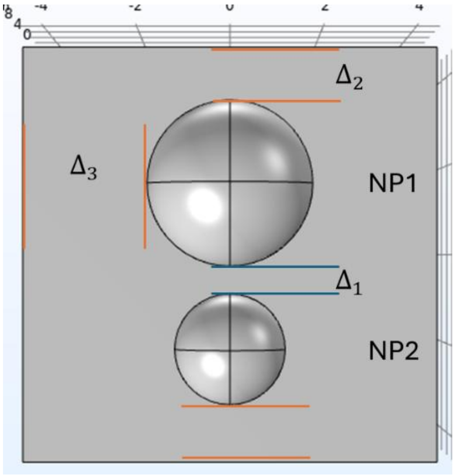

$\Delta_1 = 5[\mathrm{nm}]$
$\Delta_2 = 12.5[\mathrm{nm}]$
$\Delta_3 = 27[\mathrm{nm}]$
$p_x = \Delta_1 + 2 * \Delta_2 + 2 * (R1 + R2) = 90[nm]$
$p_y = 2 * \Delta_3 + 2 * \mathrm{R1} = 90[\mathrm{nm}]$
R1=18[nm]
R2=12[nm]

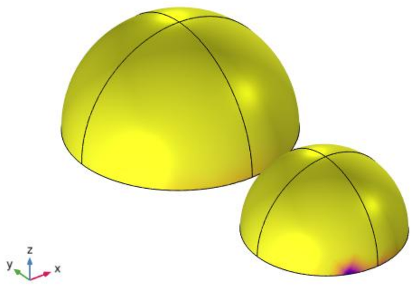

Figure S14. COMSOL models with parameters used.

11. Experiment with colloidal solution of Ag NPs

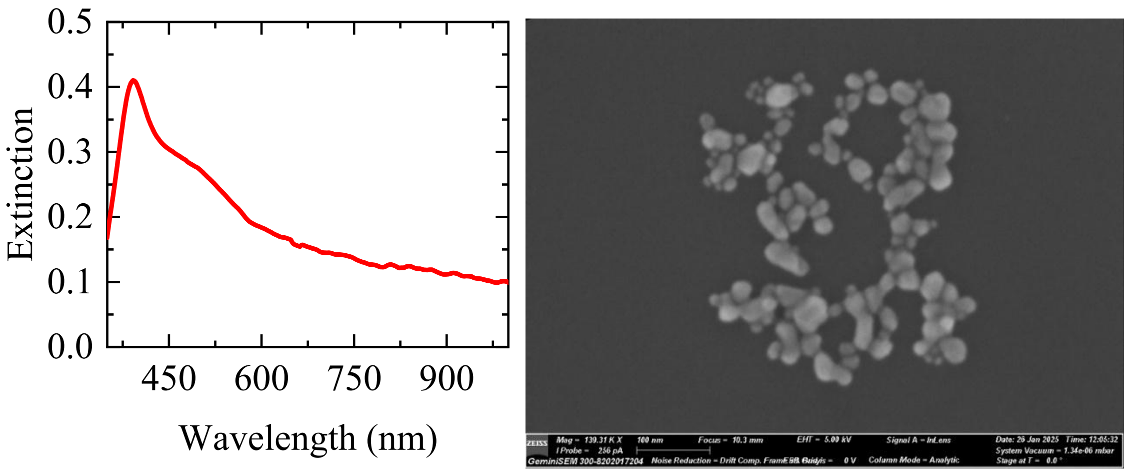

Figure S15. The extinction spectrum of colloidal Ag NPs used in ICP-MS experiment and corresponding SEM image.

12. XPS data

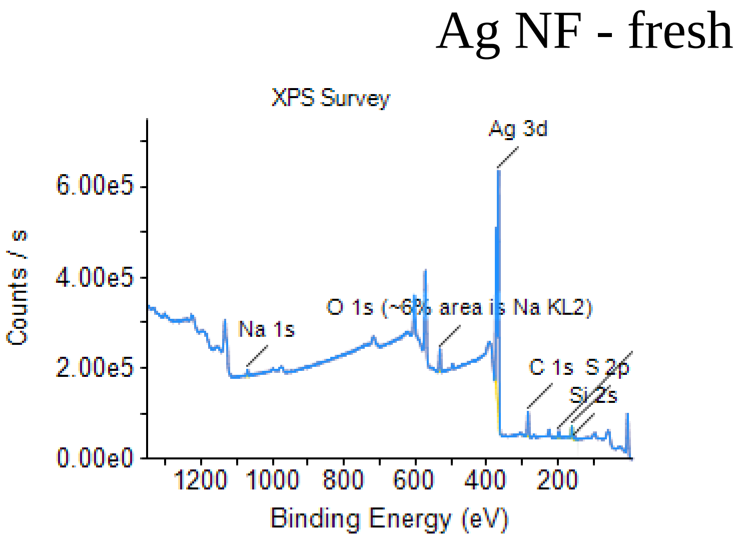

Figure S16. XPS spectrum of fresh Ag NF.

Table S2. Surface elemental composition of the fresh Ag film determined by XPS analysis.

Name	*Peak BE*	*FWHM eV*	*Area (P) CPS.eV*	*Atomic %*
C 1s	284.00	3.98	227848.22	39.19
Ag 3d	367.21	3.91	3478828.15	28.26
O1s	531.55	4.18	219647.46	15.13
S 2p	160.72	3.61	98359.07	8.47
Cl 2p	197.46	4.26	61256.95	3.68
Si 2s	152.67	3.89	19675.38	3.28
Na 1s	1070.20	3.98	63354.55	1.98

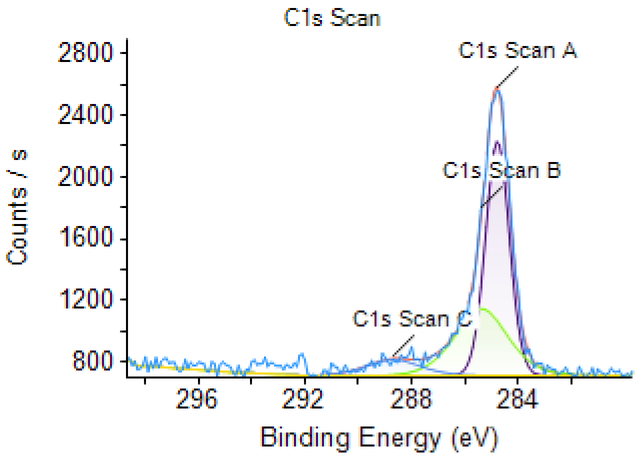

Figure S17. XPS spectrum of fresh Ag NF.

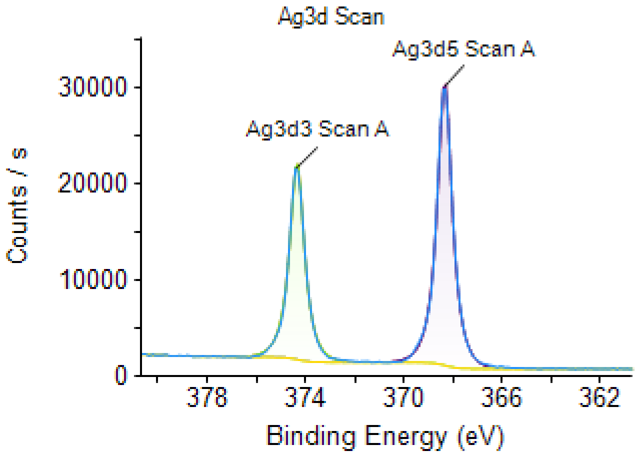

Figure S18. XPS spectrum of fresh Ag NF.

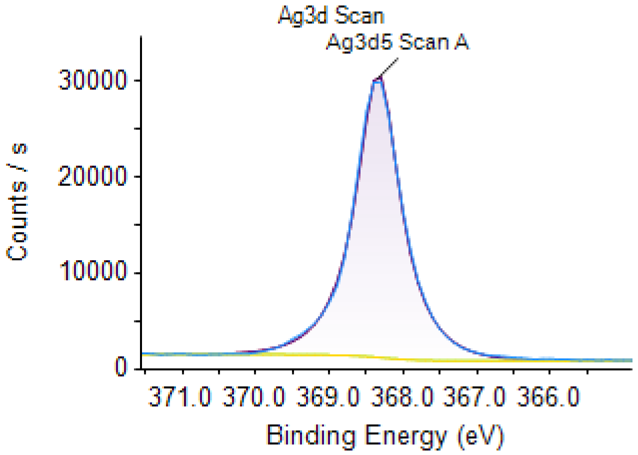

Figure S19. XPS spectrum of fresh Ag NF.

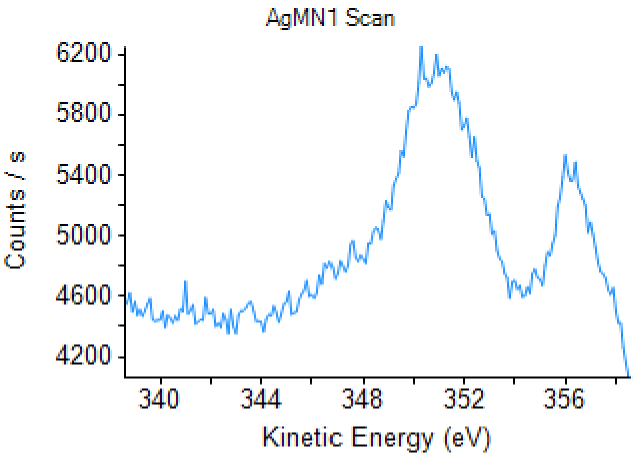

Figure S20. Auger spectrum of fresh Ag NF.

Auger parameter = 368.3 eV + 351.6 eV = 719.9
Ag metal.

Ag NF - aged

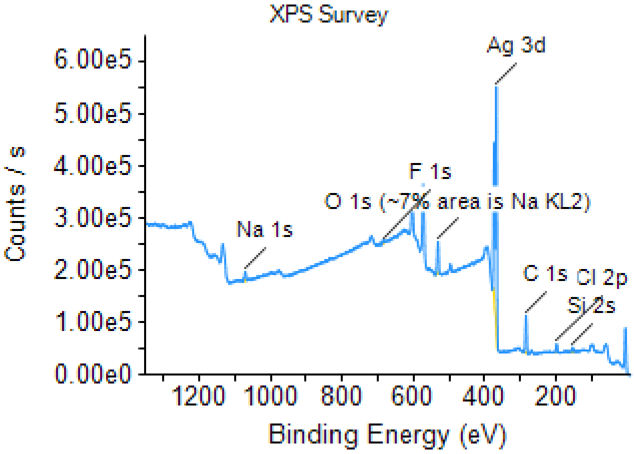

Figure S21. XPS spectrum of aged Ag NF.

Table S3. Surface elemental composition of the aged Ag film determined by XPS analysis.

Name	*Peak BE*	*FWHM eV*	*Area (P) CPS.eV*	*Atomic %*
C 1s	284.23	3.97	294365.57	46.92
Ag 3d	367.94	3.92	2979404.37	22.44
O1s	531.61	4.16	276309.95	17.63
Si 2s	153.21	3.79	36375.07	5.63
Cl 2p	198.18	4.10	70277.69	3.92
Na 1s	1071.12	4.53	85017.38	2.46
F 1s	684.87	2.30	20285.42	1.01

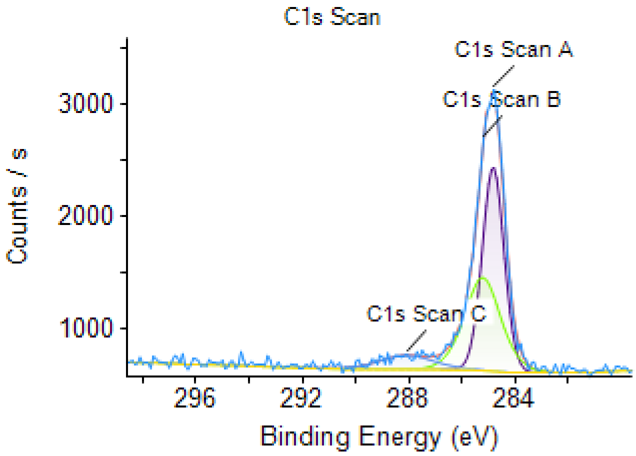

Figure S22. XPS spectrum of aged Ag NF.

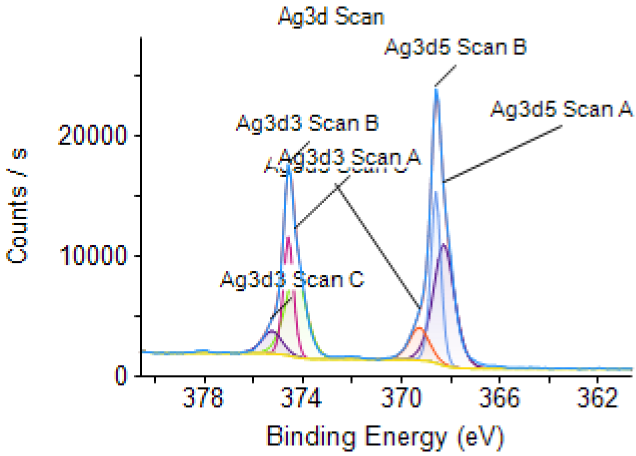

Figure S23. XPS spectrum of aged Ag NF.

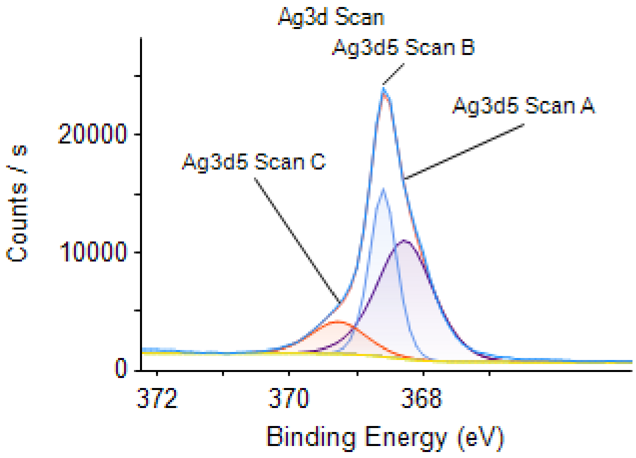

Figure S24. XPS spectrum of aged Ag NF.

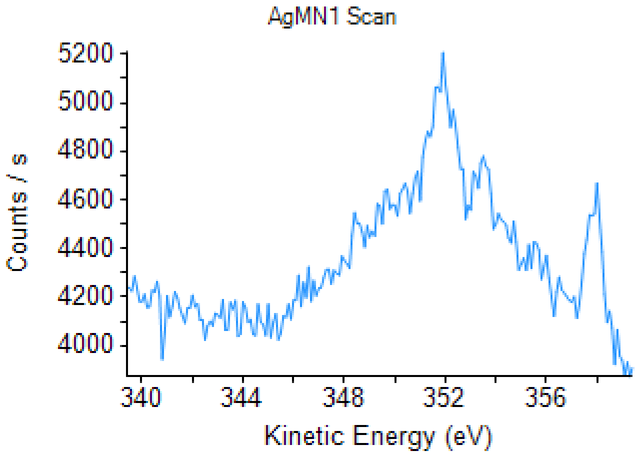

Highly mixed states of Ag, including metal and Ag+1.
Figure S25. Auger spectrum of aged Ag NF.
